# Determination of salt contents of bread types and estimation of salt intake from bread in Lebanon

**DOI:** 10.1371/journal.pone.0325857

**Published:** 2025-06-12

**Authors:** Nathalie Barakat, Ammar Olabi, Lara Nasreddine, Hussain Ismaeel, Samer Kharroubi, Layal Abou Jaoude, Mona Zeidan, Caroline Rajeh, Imad Toufeili

**Affiliations:** 1 Department of Nutrition and Food Sciences, American University of Beirut, Beirut, Lebanon; 2 Department of Internal Medicine, University of Balamand, Beirut, Lebanon; State University of Bangladesh, BANGLADESH

## Abstract

**Background:**

High dietary salt intake is a major risk factor for hypertension, which strongly predisposes affected individuals to cardiovascular diseases and stroke. Most populations consume more salt than the upper limit set by the WHO at 5 g/day. Bread is a major contributor to salt intake, and reducing bread salt is the most effective approach for reducing the ingestion of salt by populations.

**Aims:**

This work aims to determine the salt levels of bread marketed in Lebanon, bread consumption by the Lebanese population, and the bread’s contribution to daily salt intake.

**Methods:**

One hundred and sixty-two samples of the breads consumed in Lebanon were collected from 45 bakeries, and their salt levels were determined by atomic absorption spectrophotometry. The bread consumption was estimated from a cross-sectional survey of 1048 individuals, and their salt intakes were computed using the determined levels of bread salt. The proportion of breads samples meeting the WHO-recommended salt levels was computed, and the salt intakes were determined and benchmarked against the WHO cut-offs.

**Results:**

The least salty and saltiest breads were the white pita and markouk, with mean salt levels of 1.46g/100g and 2.77g/100g, respectively. The breads meeting the WHO-recommended salt levels ranged between 7.1% and 12%. The total bread consumption was 176.27 ± 216.73 g/day with white pita being the most consumed at 96.63 ± 175.44 g/day. The salt intake from bread at 2.86 ± 3.83 g/day amounted to 57.2% of the WHO limit for daily salt intake.

**Conclusions:**

The breads spanned wide ranges of salt content and differed markedly in their contribution to salt intake. White pita was the most consumed and contained the least salt thereby making it the chief contributor to salt intake from bread. Interestingly, the analyzed breads indicated the availability of products that meet the WHO-recommended targets for salt thereby providing an impetus for reducing bread salt by stealth.

## Introduction

High blood pressure is a major risk factor for ischemic heart disease (IHD) and stroke, which have remained the leading causes of death in the last 19 years [1]. In Lebanon, IHD and stroke ranked first and second among causes of death in 2019, with increases of 20.1% and 18.3% from 2009, respectively [2]. Further, the prevalence of high blood pressure in Lebanon has been reported at 61.7% in individuals aged ≥ 45 years, and with 19.3% of undiagnosed cases [3]. Amongst the modifiable risk factors for high blood pressure, dietary salt intake is considered of major importance [4].

In common with populations subsisting on Western-type diets, most of the salt ingested in Lebanon is derived from processed foods. Further, of the processed foods, bread is the main contributor to salt intake in Lebanon [5]. In breadmaking, salt is a prime functional ingredient at the dough stage due to its ability to strengthen the gluten network, decrease the stickiness of the dough, and control the activity of yeast during fermentation. Salt is also the chief determinant of bread flavor and the final product’s shelf life [6]. The diverse functionality of salt, coupled with its low cost, is largely responsible for the extensive range of salt levels used to produce bread and related baked products [6].

Several flat and French-type breads are produced and marketed in Lebanon. Like other countries in the Middle East, bread is an integral part of the diet in Lebanon. The flatbreads are normally cut and shaped into scoops to carry foods into the mouth or are topped with food and rolled into cylinder-like sandwiches. The French baguettes are normally split, filled with food, and consumed as sandwiches, typically by children and young adults. A national survey conducted in 2007/2008 estimated a bread intake of 136.8 g/person/day [5].

The deleterious effects of excessive salt intake on health prompted the WHO to recommend a sodium intake of 2 g/day, equivalent to 5 g salt/day, to reduce blood pressure and curtail the incidence of IHD and stroke [7]. Mean sodium intakes of 2.9–3.13 g/day have been reported for Lebanon [5,8], with more than 60% of the population consuming more than the upper limit of 2 g/day for sodium.

The significant contribution of bread to the high salt intakes worldwide triggered concerted efforts by academia, bakery associations, and health agencies to reduce the salt levels in this class of baked products. To this end, salt levels in bread were determined in different countries, low-salt substitutes were identified, and techniques to amplify the salty sensation of sodium chloride were developed [6]. Further, salt reduction initiatives in the bakery industry significantly reduced salt levels of bread in many countries [9]. Furthermore, maximum salt levels in bread ranging between 0.5 and 0.82 g/100g have been recommended by different countries [10].

The universal drive towards reducing salt intake triggered efforts to lower sodium levels in processed foods, including bread, in Lebanon [5]. Within this context, the approach has been envisaged to comprise awareness campaigns on the deleterious health effects of excessive salt intake, partnerships with the bakery industries to reduce salt levels in bread, and proposing recommendations to the relevant health agencies for enacting regulations on target levels of salt for use in breadmaking. Accordingly, and in an attempt to inform these activities, the objectives of the present work are to determine a) the salt levels of breads produced in Lebanon and b) the intake of salt from bread by the Lebanese population.

## Materials and methods

### Bread samples

A large number of bakeries, which differ in size and the types of bread, operate in Lebanon. Of these, 140 bakeries get their approved share of flour through the Ministry of Economy and Trade. Analysis of the different types of bread produced by such a relatively large number of bakeries is not a trivial task, and, accordingly, breads were sampled from bakeries with a processing capacity of ≥ 80 metric tons of flour/month. Bread samples were obtained from 45 bakeries with the aforementioned processing capacity and operating across Lebanon; the samples comprised white pita (34 samples), brown pita (25 samples), white French baguette (28 samples), brown French baguette (22 samples), markouk (28 samples), and tannour (25 samples). Apart from the baguette samples, which are normally sold by the loaf, the other types of bread are packaged in polyethylene bags for display and purchase by consumers. One retail unit, containing 4–6 loaves depending on the bread type, and 3 French-baguette loaves, was obtained from the different bakeries. The most consumed type of bread in Lebanon is pita bread, and its sales are regulated by the State, which requires the sales unit to comprise 6 loaves. The samples were brought to the laboratory in polyethylene bags and stored at −15 °C until analyzed.

### Chemical analyses

For each brand, the bread samples were defrosted, and one loaf was removed, at random, from the retail units of all bread types except the French baguette types, where one loaf was taken, at random, from the three purchased loaves.

The bread loaves were torn quickly into pieces (~2 × 3 cm), and three subsamples were placed in aluminum dishes for moisture determination according to AACC method 44–15.02 [11]. The dried samples were ashed at 500 °C for 16 h, and their sodium contents were determined by atomic absorption spectrophotometry (SOLAAR Atomic Absorption Spectrophotometer, Thermo Labsystems, MA, U.S.A.) according to AACC method 40–71 [11]. The sodium content per 100 g of fresh bread was computed by reference to the determined moisture content, and the equivalent sodium chloride content was obtained by multiplying the sodium level by 2.54 “S1 File”. The veracity of the analytical determinations was checked by analyzing standard reference materials (SRMs) (non-fat milk powder, SRM 1549 and bovine liver, SRM 1577c; National Institute of Standards & Technology, Gaithersburg, MD, USA) for sodium and spiking bread samples with standard sodium solutions (2 mg, 4 mg, and 6 mg) to assess the influence of the sample matrix on sodium recovery. Analysis of the SRMs yielded average values for replicate determinations of sodium of 496 ± 7.77 mg/100g (C.V. 1.57%) for non-fat milk powder and 198.8 ± 4.50 mg/100g (C.V. 2.26%) for the bovine liver as compared to the certified values of 497 mg/100g and 203.3 mg/100g, respectively. Replicate analysis of the spiked bread samples with sodium yielded recoveries of 96.4%, 97.4%, and 96.3% for the bread samples spiked with 2 mg, 4 mg, and 6 mg of sodium, respectively. The determined values of sodium for the SRMs and the high recoveries registered for the spiked samples indicate that the analytical protocol has the requisite accuracy and reliability for sodium determination of bread.

The Shapiro-Wilk and Kolmogorov-Smirnov tests returned significant deviations from normality (*P* ≤ 0.002) for the salt contents of white pita and markouk bread.

### Dietary intake of salt from bread

A cross-sectional survey of adult supermarket shoppers was conducted from 17/09/2014-07/08/2015. Data collection was performed in three Lebanese governorates (Greater Beirut, North, and Mount Lebanon). The collection of data in the three other governorates of Lebanon was hampered by security clearance challenges, given that access to these governorates is controlled by tight security measures. In each governorate, data collection was conducted by trained researchers in different districts and supermarkets that were chosen randomly. Data collection was performed on different days of the week, including weekends, and at different times of the day. Adult subjects were invited to participate based on the following inclusion criteria: (1) Lebanese nationality, (2) consumers of bread, and (3) 18 years and older. Systematic random sampling was adopted in each supermarket, with the sampling interval set at four. The researchers, positioned at the entrances of the supermarket, approached every fourth passing shopper and invited him/her to participate in the study [12]. In the case where the shopper refused to participate, the researcher approached the next passing shopper. Oral consent was obtained before enrolling in the study. The study design, recruitment methodology, and consenting process were approved by the Institutional Review Board of the American University of Beirut. The study did not include individuals less than 18 years old, and the data were collected anonymously and without any recognized identifiers.

A questionnaire was administered in an interview setting to each participating subject to obtain information on socio-demographic characteristics (age, gender, marital status), education level, employment status, and crowding index as an indicator of socioeconomic status [13]. Bread consumption was assessed using a checklist that inquired about the consumption of various types of bread, the frequency of consumption (per day, week, month, or never), and the portions consumed in reference to pictures of standard serving sizes. Information about the specific brands of bread that the subjects usually consume was also obtained.

Individuals’ salt intake was computed by combining consumption data (g/day) for each bread type with the salt content of the usually consumed brand (g/100g); the average salt content of the bread type was used when the subject did not report brands or reported brands that were not analyzed for their salt content in the present work. This was followed by summing up the results over the various types of bread “S2 File”.

The Shapiro-Wilk and Kolmogorov-Smirnov tests showed significant deviations from normality (*P* ≤ 0.001) for bread consumption and sodium intakes from all bread types.

### Statistical analysis

The normality of the data on salt levels of bread types, as well as bread consumption and intakes of salt from the different breads, was checked by the Shapiro-Wilk and Kolmogorov-Smirnov tests.

Differences among consumption of bread types and the salt contents and salt intakes from the different bread types were ascertained by the Kruskal-Wallis test, and means were separated by Dunn’s test with Bonferroni correction when differences were significant [14].

Since there is no recommended level of salt in bread in Lebanon, the salt contents of the pita-type bread were compared to 0.5 g salt/100g bread and the other types of bread to 0.83 g salt/100 g bread [10]. The mean salt levels of the different bread types were compared to the indicated levels by the Mann-Whitney test, and the proportions of bread not complying with the cut-offs were computed.

Statistical tests were carried out on the Statistical Package for Social Sciences (SPSS) [15]

## Results and discussion

### Salt content of bread

The pita-type breads contained significantly lower salt levels (*P* < 0.05) than the other breads, and the brown baguette and tannour had significantly less salt (*P* < 0.05) than the markouk bread (Table 1). No differences (*P *> 0.05) were detected between the white and brown versions of pita or baguette bread, between white baguette and markouk or tannour, and between brown baguette and tannour bread (Table 1). The lowest mean salt level was registered for the white pita-type bread at 1.46g/100g, while the saltiest bread was the markouk-type with an average salt content of 2.77g/100g (Table 1). The different salt levels observed for the different bread types reflect their different formulations, the required strength of the gluten network at the dough stage, and intended storage. It is noteworthy that tannour and markouk breads were traditionally the mainstays of people in the arid regions of the Fertile Crescent [16] when refrigeration facilities were not available, and the keeping quality of the breads, at the high ambient temperatures, was essentially determined by their high salt levels. The average salt contents of white pita, markouk, and tannour types of bread in the present study were higher than those reported for the generically similar white Arabic bread (0.42g/100g), shrak (2.06g/100g) and mashrouh (1.61g/100g), respectively [17]. Wide ranges of salt contents were found for the different bread types surveyed in Nigeria [18], Mozambique [19], and Iran [20]. Further, wide ranges of salt contents have been reported for the same type of bread within a geographical region and across regions from Morocco [21], the Republic of Srpska [22], and Mozambique [23]. The wide ranges of salt added to bread are primarily intended to optimize the dough quality from flours of different strengths and to meet the diverse preferences and salt sensitivities of the different segments of the population [6].

**Table 1:** Salt contents (g/100g) and proportion of bread samples conforming to the World Health Organization (WHO) guidelines for bread types marketed in Lebanon.

Bread type	Number of samples	Salt (g/100g) Mean ± SD Range	% Products on target^e^
White pita	34	1.46 ± 0.85^a^ 0.17–5.10	8.8
Brown pita	25	1.52 ± 0.80^a^ 0.11–3.37	12.0
White Baguette	28	2.24 ± 0.89^b^ 0.38–3.95	7.1
Brown Baguette	22	2.05 ± 0.89^bc^ 0.49–4.30	9.1
Markouk	28	2.77 ± 1.05^bd^ 0.70–4.39	7.1
Tannour	25	2.18 ± 0.89^bc^ 0.17–3.94	8

a, b, c, d . Means with different superscripts are significantly different (*P* < 0.05) by the Dunn-Bonferroni test.

e . Salt levels of white and brown pita were compared to 0.5 g/100 g and those of the baguette-type, markouk, and tannour to 0.83g/100g [10]. All means were significantly different from the target level (*P* < 0.000) by the Mann-Whitney test.

The mean salt levels of the pita breads were compared to 0.5g/100 g and those of the other types of bread to 0.83 g/100 g which have been set by WHO [10] for the different yeast-leavened bread. The Mann-Whitney test returned significant differences (*P* < 0.000) between the mean salt levels and 0.5g/100g for the pita-type breads, and 0.83 g/100 g for the baguette-type, markouk, and tannour (Table 1). The percentages of pita-type, baguette-type, markouk, and tannour that met the WHO cut-offs were 8.8–12%, 7.1–9.1%, 7.1%, and 8%, respectively (Table 1). The markedly low proportion of bread meeting the WHO cut-offs for salt levels of different bread types, warrants serious efforts by the different stakeholders to address the current practices of bread production, in Lebanon, including reformulation of the bake mix to contain lower amounts of salt.

### Intake of salt from bread

**Fig 1:**
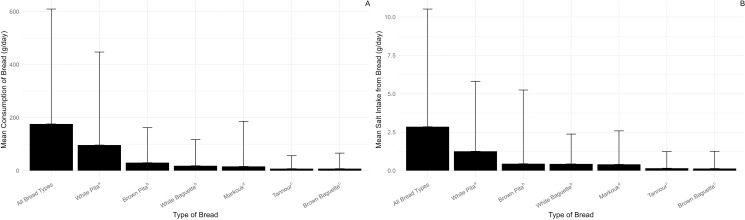
Bread consumption (A) and salt intake from bread (B) (Error bars: ± 2 SD). a, b, c, d, e indicate significant differences (*P* < 0.05) by the Dunn-Bonferroni test.

A sample of 1048 subjects (551 females and 497 males) participated in the survey. The average age of the participating subjects was 35.36 ± 14.27 years, with no significant differences between males and females “S1 Table”. Of the study population, 47.2% were single, 62.3% were employed and 43.7% had a crowding index ≥ 1 person/room, attesting to a lower socioeconomic status [13]. The participants preferred particular bread brands and rarely bought other brands. Further, the proportions of the participants who did not indicate a brand, or purchased brands that were not analyzed in the present work, for white pita, brown pita, white baguette, markouk, and tannour were 21.1%, 15.6%, 15.5%, 10.9%, 14.7%, and 13.1%, respectively. The relatively low number of missing brands indicates that the analytical data for bread salt accounted for ≥ 78.9% of salt levels ingested by the participants from bread. Fig 1 displays the consumption levels of the various types of bread and sodium intake from bread in Lebanon. The consumption of white pita was higher (*P* < 0.05) than the other bread types, markouk consumption was higher (*P* < 0.05) than those of brown baguette and tannour, and no differences were detected between brown pita and white baguette and between the brown baguette and tannour bread. The most highly consumed type of bread was white pita (96.63 ± 175.44 g/day), followed by brown pita (30.11 ± 65.92 g/day) and white baguette (18.48 ± 49.35 g/day). The total daily bread consumption was estimated at 176.27 ± 216.73 g/day, a value higher than estimates reported by previous studies conducted amongst adults in Lebanon (136.8–146.2 g/day) [5]. Expectedly, and in line with previous findings [24,25], a significantly higher consumption level was observed in men compared to women (240.54 ± 276.04 vs. 118.31 ± 116.64 g/day; *P* < 0.001).

The mean salt intake from bread, estimated at 2.86 ± 3.83 g/day, amounted to 57.2% of the 5 g upper daily intake limit set by the WHO [10]. The observed salt intake level is higher than reported by a previous study conducted in Lebanon (1.9 g/day) which was based on food composition software rather than actual analysis of bread samples [5]. Further, of the total daily salt intake from bread (2.86 g/day), the pita type contributed 1.70 ± 2.37 g/day, the baguette type provided 0.55 ± 1.27 g/day, and the markouk and tannour breads supplied the balance of 0.62 ± 2.57 g/day (Fig 1). Notwithstanding their lowest salt levels (Table 1), the pita-type breads contributed the most to the salt intake due to them being the most consumed breads in Lebanon. This finding is shared by several countries in the Eastern Mediterranean where pita-type breads are the most consumed baked products [26]. The relatively low consumption of baguette-type breads in households was largely responsible for their low contribution (0.55 ± 1.27 g/day) to the salt intake from bread, notwithstanding their high salt contents (Table 1). This finding might be attributed to the preferential use of baguette-type breads in sandwich-making by fast-food and takeaway outlets in the country, and, accordingly, their contribution to salt intakes is expected to be higher than determined in the present work. The tannour and markouk breads are widely considered rustic and universally perceived as being healthier than the mass-produced pita-type bread. However, their high salt levels (Table 1) are incongruent with their perceived healthfulness, and their consumption ought to be reduced by the general population, especially hypertensive individuals. Gender-based differences were observed in our study with a significantly higher intake of salt in men compared to women (3.86 ± 4.99 vs. 1.97 ± 1.93 g/day; *P* < 0.001). The higher salt intake by men has been attributed to their higher energy intake and preference for foods containing large amounts of salt [27]. These findings suggest that adult men may reach 77% of the WHO upper limit (UL) from bread consumption alone and are therefore at a high risk of exceeding this UL daily. The salt intake from bread in Lebanon (2.86 g/day) was higher than estimates reported from Mexico (0.62g/day) [28], Ireland (0.84g/day) [29], U.K. (1.2 g/day) [30], and Serbia (1.7g/day) [31] and lower than Tunisia (3.7g/day) [32] and Morocco (8.7 g/day) [21].

In its report on sodium intake reduction, the WHO outlines multifaceted initiatives that its 194 member states have adopted to reduce the salt intake of populations. These initiatives include a) policies for purchasing, distribution, and sales of low-salt foods in public settings comprising schools, hospitals, and government workplaces, b) information on salt contents in the nutrition labels, c) media campaigns to increase awareness of the harmful effects of high salt intake to affect behavioral change in consumers, and d) reformulation of foods that contribute most to dietary salt intake to reduce their salt levels [33]. Broadly similar policies for salt reduction have been championed by the Lebanese Action on Sodium and Health (LASH) to ward off the increasing prevalence of hypertension in the nation [5]. The findings of the present work could potentially inform the aforementioned policies by providing information on the population’s salt intake and salt levels of bread for use in procurement policies and serving of healthy foods, media campaigns for improving the dietary habits of the population, and the development of nutrition labels for bread. Analysis of the relative contributions of the different policy components to salt intake reduction indicates that reducing the salt levels of foods by reformulation is the most effective approach for lowering the population’s salt intake [34]. The effectiveness of reformulation, especially when coupled with mandatory setting of maximum salt levels, in lowering populations’ salt intake has been attributed to the creation of marketplaces that limit unhealthy product options, persuasion of the food industry to develop healthier product analogs, and obviating the need for action from consumers [33]. Given its significant contribution to populations’ salt intake, bread has been the most targeted product for reformulation in the Eastern Mediterranean, including Lebanon [26]. The present work showed that pita bread is the most-consumed bread and the chief contributor to salt intake from bread (Fig 1). Further, the salt levels of pita bread spanned a wide range (Table 1) thereby indicating that production of this type of bread with varied salt levels is technically feasible, and analogs of pita bread with salt levels of 0.5 g/100g, as recommended by the WHO [10], are being traded in the Lebanese market. These findings lend a strong impetus to reformulating pita bread to the recommended 0.5g salt/100g advocated by WHO [10]. Several approaches have been utilized to lower the sodium levels of bread, including using salt substitutes, amplifying the salty sensation of sodium chloride through contrast and/or modification of sodium chloride crystal size and structure, and gradually reducing salt levels [6]. A 50% reduction in sodium chloride through substitution with potassium chloride has been achieved in pan bread without affecting the consumer acceptability of the product [35]. Likewise, a 28% reduction in sodium has been realized in baguette-type bread through substitution with potassium citrate, as evidenced by the little awareness of the reduced salt levels in market studies in Spain [36]. The heterogeneous spatial distribution of sodium chloride resulted in a reduction of 33% in salt levels in bread rolls without perceptible changes in the saltiness intensity of the product [37]. The use of coarse-grained sea salt (2–3.5 mm) and salt microspheres (~ 600 µm), in bread formulation allowed for a reduction of 25% in salt in pan bread and white pita bread, respectively [38,39]. Gradual incremental reduction in salt levels by stealth reduced the salt contents in Danish and French bread by 33–35% without affecting the consumer acceptability of the product [32,40]. Over time, the approach involving gradual small reductions in salt levels has been particularly effective in reducing the sodium contents of foods including bread in several developed and developing countries. Further, for successful implementation, the strategy of gradual reduction of sodium levels is executed in combination with setting national targets for salt levels, collaborating with the baking industry, and conducting awareness campaigns on the deleterious effects of high salt intakes on health. To this end, a 20% reduction in salt levels of bread (from 1.23g/100g to 0.98g/100g) has been achieved between 2001 and 2011, in the UK, with a concomitant increase in the percentage of breads meeting the Food Standards Agency targets of 0.98 g salt/100 g bread from 25% to 71% [41]. A similar trend has been reported in New Zealand with a 6% reduction in bread sodium (from 469 mg/100g to 439 mg/100g) between 2007 & and 2010 with an increase in the percentage of breads meeting the standard of 450 mg Na/100g of bread from 49% in 2007 to 90% in 2010 [42]. Of note, the targeted lower salt levels of foods, including bread, should be rigorously regulated because the availability of products with higher salt levels decreases the acceptability of the reduced-salt versions [40]. In this connection, the effectiveness of reformulating foods, including bread, improved significantly when coupled with setting mandatory, rather than voluntary, salt targets [34]. The reduction in salt levels in bread and its contribution to salt intake in England has been suggested as a significant factor in the observed decrease in blood pressure from 2003 to 2011 [43].

The present work has strengths and limitations that must be considered when interpreting its findings. A major strength of the study is the analysis of the salt content of bread brands from bakeries with a processing capacity of ≥ 80 tons of flour/month, which captured almost 79% of the variation in the salt intake. However, the exclusion of breads produced by bakeries with lower processing capacities is a limitation of the study because their analysis would have provided a holistic assessment of bread salt levels marketed in the country. In addition, as with many questionnaire-based surveys, the interview-based data collection method may have introduced a social desirability bias, whereby respondents provide answers they believe are acceptable or favorable to the interviewer [44]. To minimize this bias, the fieldworkers who collected the data were extensively trained on reducing judgmental verbal and nonverbal communication.

With the availability of analytical data on the salt content of national bread types, it is recommended that LASH move forward with involving the private sector in reformulating bread to the WHO-recommended salt levels and developing effective media campaigns to spread awareness on the deleterious health effects of high salt intakes. It is also recommended that LASH lobby the parliament to set mandatory salt levels for pita and the other types of bread and enact regulations for mandatory front-of-pack labelling of food products. It is further recommended that analytical data for salt be determined for dairy products, and processed meat and poultry products, which have been reported to contribute significantly to the national salt intake [5].

## Conclusions

Samples of the bread types consumed in Lebanon were analyzed for their salt content, and the generated data were used to estimate the population’s salt intake from bread. The analytical determinations covered 78.9–88.1% of the salt levels of bread types traded in the country. The data showed wide salt ranges within and among the different bread types. The computed salt intakes using the analytical data were higher than those estimated in studies using nutrition composition software for calculating the average salt contents of food commodities. The white pita was the least salty and most consumed, contributing most to the salt intake from bread given its disproportionately high consumption. The mean salt intake from bread amounted to 57.2%, and reached 77% in males, of the WHO upper limit for salt intake (5g/day). The analytical data on bread salt and salt intake from bread have potentially pivotal roles in informing policies aimed at reducing dietary salt intake of populations comprising the use of low-salt foods in public settings, media campaigns on the negative health effects of excessive salt intakes, developing nutrition labels for bread, and collaborating with the baking industry for reformulating bread to contain lower levels of salt.

## Supporting information

S1 File Salt levels of breads. Salt contents (g/100g) of bread types consumed in Lebanon.
(XLSX)

S2 File Salt intake from breads. Population Descriptives, bread consumption (g/day) and salt intake (g/day) from bread in Lebanon.
(XLSX)

S1 Table Sociodemographic characteristics, bread consumption, and salt intake (Mean ± SD) of the study population by gender. (DOCX)
