# Supplementary material for: Determination of salt contents of bread types and estimation of salt intake from bread in Lebanon
Source: PLoS One. 2025 Jun 12;20(6):e0325857. doi: 10.1371/journal.pone.0325857 (PMC12161562; doi:10.1371/journal.pone.0325857)
Supplement: S1 Table — (DOCX) [file pone.0325857.s003.docx]

Sociodemographic characteristics, bread consumption, and salt intake (Mean ± SD) of the study population by gender.

| **Variable** | **Total (n = 1048)** | **Males (n = 497)** | **Females (n = 551)** | **p-value** |
| --- | --- | --- | --- | --- |
| Age | 35.36 ± 14.27 | 35.67 ± 14.76 | 35.08 ± 13.82 | 0.730^a^ |
| Educational Level  No schooling  Elementary  Middle school/primary  High school/secondary  University | 29 (2.8%)  56 (5.3%)  95 (9.1%)  176 (16.8%)  691 (65.9%) | 12 (2.4%)  31 (6.2%)  47 (9.5%)  83 (16.7%)  324 (65.2%) | 17 (3.1%)  25 (4.5%)  48 (8.7%)  93 (16.9%)  367 (66.7%) | 0.721^b^ |
| Family Status  Single/never married  Married  Divorced  Widowed | 495 (47.4%)  480 (45.9%)  39 (3.7%)  31 (3.0%) | 251 (50.7%)  210 (42.4%)  20 (4.0%)  14 (2.8%) | 244 (44.4%)  270 (49.1%)  19 (3.5%)  17 (3.1%) | 0.169^b^ |
| Work Status  Employed^1^  Housewife^2^  Retired  Unemployed  Student | 653 (62.4%)  153 (14.6%)  39 (3.7%)  42 (4.0%)  160 (15.3%) | 376 (75.8%)  6 (1.2%)  23 (4.6%)  24 (4.8%)  67 (13.5%) | 277 (50.3%)  147 (26.7%)  16 (2.9%)  18 (3.3%)  93 (16.9%) | <0.001^b^ |
| Crowding Index | 1.02 ± 1.31 | 0.99 ± 0.68 | 1.05 ± 0.07 | 0.623^a^ |
| Bread Consumption (g/day)  White pita^3^  Brown pita^3^  White baguette  Brown baguette^3^  Markouk  Tannour | 176.27 (±216.73)  96.63 ± 175.44  30.11 ± 65.92  18.48 ± 49.35  7.49 ± 29.34  16.04 ± 85.15  7.52 ± 24.35 | 240.54 (±276.04)  147.03 ± 227.15  31.12 ± 73.18  23.27 ± 60.65  7.83 ± 31.51  23.00 ± 120.42  8.29 ± 27.49 | 118.31 (116.64)  51.17 ± 87.72  29.20 ± 58.66  14.17 ± 35.76  7.18 ± 27.27  9.76 ± 25.36  6.84 ± 21.13 | <0.001^a^ |
| Salt intake (g/day)  White pita^4^  Brown pita^4^  White baguette  Brown baguette^4^  Markouk  Tannour | 2.86 ± 3.83  1.26 ± 2.28  0.44 ± 0.97  0.41 ± 1.09  0.14 ± 0.56  0.45 ± 2.40  0.16 ± 0.54 | 3.86 ± 4.99  1.91 ± 2.95  0.46 ± 1.07  0.51 ± 1.33  0.15 ± 0.60  0.65 ± 3.39  0.18 ± 0.60 | 1.97 ± 1.93  0.66 ± 1.14  0.43 ± 0.86  0.31 ± 0.79  0.14 ± 0.52  0.27 ± 0.71  0.15 ± 0.46 | <0.001^a^ |

^a^Mann-Whitney U Test, ^b^Pearson Chi-Square Test, ^1^Significantly more males are employed than females, ^2^Significantly more females are housewives than males,

^3^Significanly more bread is consumed by males, ^4^Significantly higher salt intake by males.
